# Environmental neurotoxin interaction with proteins: Dose-dependent increase of free and protein-associated BMAA (β-*N*-methylamino-L-alanine) in neonatal rat brain

**DOI:** 10.1038/srep15570

**Published:** 2015-10-26

**Authors:** Oskar Karlsson, Liying Jiang, Lisa Ersson, Tim Malmström, Leopold L. Ilag, Eva B. Brittebo

**Affiliations:** 1 Department of Pharmaceutical Biosciences, Uppsala University, SE-751 24 Uppsala, Sweden; 2 Department of Environmental Science and Analytical Chemistry, Stockholm University, SE-10691, Stockholm, Sweden.

## Abstract

β-Methylamino-*L*-alanine (BMAA) is implicated in the aetiology of neurodegenerative disorders. Neonatal exposure to BMAA induces cognitive impairments and progressive neurodegenerative changes including intracellular fibril formation in the hippocampus of adult rats. It is unclear why the neonatal hippocampus is especially vulnerable and the critical cellular perturbations preceding BMAA-induced toxicity remains to be elucidated. The aim of this study was to compare the level of free and protein-associated BMAA in neonatal rat brain and peripheral tissues after different exposures to BMAA. Ultra-high performance liquid chromatography-tandem mass spectrometry analysis revealed that BMAA passed the neonatal blood-brain barrier and was distributed to all studied brain areas. BMAA was also associated to proteins in the brain, especially in the hippocampus. The level in the brain was, however, considerably lower compared to the liver that is not a target organ for BMAA. In contrast to the liver there was a significantly increased level of protein-association of BMAA in the hippocampus and other brain areas following repeated administration suggesting that the degradation of BMAA-associated proteins may be lower in neonatal brain than in the liver. Additional evidence is needed in support of a role for protein misincorporation in the neonatal hippocampus for long-term effects of BMAA.

Non-protein amino-acids are synthesized by plants or microorganisms as nitrogen storage or biological defence since some of them can act as antimetabolites in exposed competing organisms^1^. β-Methylamino-*L*-alanine (BMAA) is a non-protein amino acid and neurotoxin that is produced by the cosmopolitan organisms cyanobacteria and diatoms^2^,^3^. BMAA has been suggested to be associated with the rare medical condition amyotrophic lateral sclerosis and Parkinsonism-dementia complex (ALS/PDC) on islands in the Western Pacific region^4^,^5^. This neurotoxin is detected in aquatic ecosystems, including in molluscs, crustaceans and fish that are consumed by humans, which indicates that BMAA bioaccumulates in aquatic food chains^6^,^7^,^8^,^9^. BMAA has also been reported to be present in brains of Alzheimer’s disease patients and ALS patients^10^,^11^, but so far the analytical data are conflicting^12^,^13^. BMAA is a developmental neurotoxin^14^,^15^,^16^ that induces long-term cognitive deficits as well as protein changes and fibril formation in the hippocampus of adult rodents following neonatal exposure^17^,^18^,^19^,^20^,^21^. The critical cellular perturbations preceding BMAA-induced intracellular fibril formation in the hippocampus remains to be elucidated. Interestingly, enrichment of proteins implicated in protein aggregation, and an increased protein ubiquitination was observed in the adult hippocampus following neonatal administration^19^,^20^,^21^. This may indicate that the non-protein amino acid BMAA could contribute to protein misfolding and/or aggregation that are hallmarks of many neurodegenerative disorders.

Our previous autoradiographic imaging studies of radiolabelled BMAA in adult and neonatal rodents revealed a tissue distribution pattern similar to protein-forming amino acids, indicating that BMAA may be incorporated or associated with newly synthesized proteins^22^,^23^,^24^,^25^. In addition, ultra-high performance liquid chromatography-tandem mass spectrometry (UHPLC-MS/MS) analysis revealed a dose-dependent increase of protein-associated BMAA in the neonatal liver and to a lower extent in brain regions not fully protected by the blood-brain barrier^25^. Other reports have also indicated that BMAA may be associated with or incorporated into proteins in animals and cultured cells^26^,^27^,^28^,^29^,^30^,^31^. In contrast, no protein-association was detected in BMAA-exposed zooplankton grazer *Daphnia magna* although free BMAA was detected in this species^32^. The aim of this study was to characterize the tissue distribution of free and protein-associated BMAA following various doses and exposures in neonatal rats using UHPLC-MS/MS with special reference to the BMAA-induced long-term damage in the adult hippocampus of neonatally exposed rats.

## Results

### Identification of BMAA, isomers and limit of detection (LOD) and limit of quantitation (LOQ) for the analytical method

The LOD and LOQ for the analytical method were estimated to be below 0.01 ng BMAA/mg wet weight of brain tissue (based on S/N > 3 for transition 459.18 > 258.09 and S/N > 10 for transition 459.18 > 119.08, respectively). The BMAA isomer *N*-(2-aminoethyl) glycine (AEG) was detected in the protein fraction in some samples at a very low concentration, while other isomers, 2, 4-diaminobutyric acid (DAB) and β -amino-N-methyl-alanine (BAMA) were undetectable in all samples.

### Quantification of free BMAA in the tissues

Free BMAA in neonatal rats given BMAA (2 × 40 mg/kg) on PND 9 and 10 was detected in all analysed tissue samples (Table 1). The highest concentration of free BMAA was detected in the liver and the skeletal muscle. In the central nervous system the level of free BMAA was similar in all studied brain areas (i.e. cortex, hippocampus, striatum and cerebellum) whereas the hypothalamus had a higher level. Tissues with a high cell turnover such as the thymus, spleen and pancreas had relatively low concentrations of free BMAA. A dose-dependent increase of free BMAA was detected in all analysed tissues of neonatal rats given BMAA (2 × 40 mg/kg or 2 × 150 mg/kg) on PND 9 and 10. The highest increase (20-fold) was observed in the hippocampus. In animals given five repeated injections of BMAA on PND 9-13 (5 × 40 mg/kg), most tissues had a somewhat increased tissue concentration of free BMAA in comparison with animals administrated BMAA for two days with the exception of the skeletal muscle that had a significantly decreased level.

### Quantification of protein-associated BMAA in the tissues

**Figure 1:**
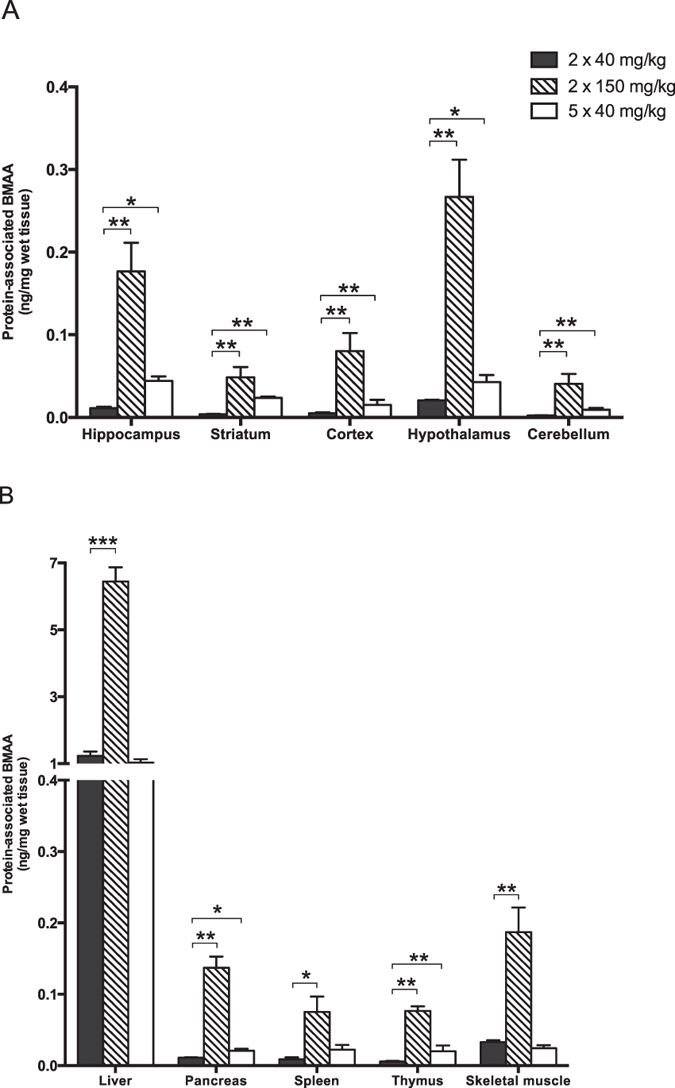
Male neonatal rats were given subcutaneous injections of BMAA. Twenty-four hours after the last injection the animals were killed and selected tissues were rapidly frozen for subsequent UHPLC-MS/MS analysis of protein-associated BMAA. The figure shows mean ± SEM. *p < 0.05, **p < 0.01, ***p < 0.001 compared with the group administered 2 × 40 mg/kg BMAA (ANOVA followed by Student's t-test test. Chi-square test was used when groups had not detectable levels of protein-associated BMAA).

Protein-associated BMAA in neonatal rats given BMAA (2 × 40 mg/kg) on PND 9 and 10 was detected in all analysed tissue samples except for striatum, cerebellum, cortex and thymus where the levels were below the LOD and LOQ for the analytical method (Table 1 and Fig. 1). The highest concentrations of protein-associated BMAA were detected in the liver and skeletal muscle. In the brain the level of protein-associated BMAA was the highest in the hippocampus and hypothalamus. A dose-dependent increase of protein-associated BMAA was detected in all analysed tissues of neonatal rats given BMAA (2 × 40 mg/kg or 2 × 150 mg/kg) on PND 9 and 10. The hippocampus showed a 16-fold increase (Table 1). The level of protein-associated BMAA in neonatal rats given five repeated injections of BMAA on PND 9–13 (5 × 40 mg/kg) was higher in most tissues, compared to the animals administered BMAA for two days, except for the liver and the skeletal muscle.

**Figure 2:**
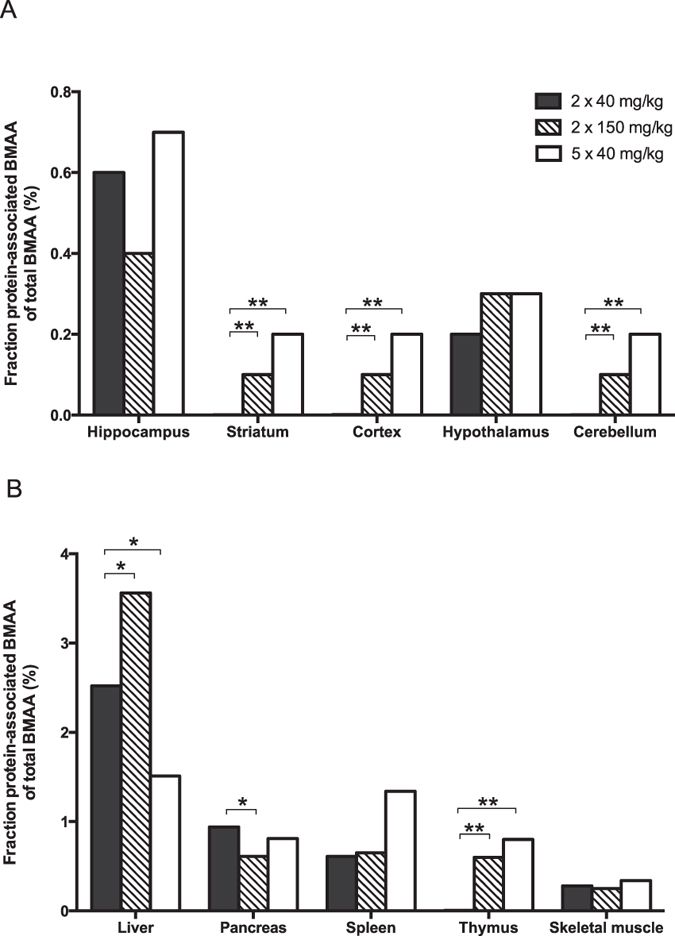
Male neonatal rats were given subcutaneous injections of BMAA. Twenty-four hours after the last injection the animals were killed and selected tissues were rapidly frozen for subsequent UHPLC-MS/MS analysis of free and protein-associated BMAA. The figure shows the percentage of protein-associated BMAA of total BMAA (protein-associated BMAA/total levels of BMAA) in neonatal rat tissues. *p < 0.05, **p < 0.01, ***p < 0.001 compared with the group administered 2 × 40 mg/kg BMAA (ANOVA followed by Student's t-test test. Chi-square test was used when groups had not detectable levels of protein-associated BMAA).

Finally, the fraction of protein-associated BMAA in relation to the total amount of BMAA in the tissues was calculated (Table 1 and Fig. 2). The results showed that the liver had the highest amount of protein-associated BMAA in relation to the total amount of BMAA in these tissues. Also the hippocampus, pancreas, spleen, and thymus had comparatively high percentage of protein-associated BMAA. All other brain areas (i.e. cortex, striatum, hypothalamus and cerebellum) and skeletal muscle had a low amount of protein-associated BMAA in relation to the total amount of BMAA in the tissues.

The calculated fraction of protein-associated BMAA in tissues of neonatal rats given BMAA (2 × 40 mg/kg or 2 × 150 mg/kg) on PND 9 and 10 showed no consistent dose-dependence. The percentage of protein-associated BMAA in hippocampus, hypothalamus, skeletal muscles and spleen was unaffected by the dose. The striatum, cortex, cerebellum, thymus and the liver demonstrated a dose-dependent increase of the amount of protein-associated BMAA, in relation to the total amount of BMAA in the tissues. In contrast, the pancreas showed a dose-dependent decrease in the fraction of protein-associated BMAA. The percentage of protein-associated BMAA in the striatum, cortex, and cerebellum of neonatal rats given five repeated injections of BMAA on PND 9-13 (5 × 40 mg/kg) was higher than that in animals given BMAA for two days. The liver was the only tissue that showed decreased percentage of protein-associated BMAA after repeated injections whereas all other tissues had similar fractions as after two injections.

## Discussion

The cyanobacterial toxin BMAA has been implicated in the aetiology of neurodegenerative disorders^4^,^11^,^33^ and the uptake and potential protein-association of BMAA in mammalian central nervous system are of concern^29^,^31^,^34^. The aim of this study was to compare the level of free and protein-associated amino acid BMAA in the central nervous system and peripheral tissues 24 hours after different exposures to BMAA in neonatal rats. We have previously reported that neonatal exposure to BMAA on PND 9–10 can induce long-term cognitive impairments and progressive neurodegenerative changes including intracellular fibril formation in the hippocampus of adult rats^19^,^21^. It is unclear why the neonatal hippocampus is especially vulnerable for BMAA but neurogenesis and synaptogenesis are higher in the developing brain than in the adult brain. The uptake and protein-association of BMAA in the neonatal hippocampus and other neonatal brain areas are therefore of special concern.

The present study revealed that BMAA was distributed to all studied neonatal brain areas and the concentration of free BMAA in the neonatal brain was markedly higher than that found in peripheral tissues such as thymus, spleen and pancreas. This in spite of the fact that the brain, with the exception of hypothalamus, is often protected from substances by the blood-brain barrier^35^. In the central nervous system the detected concentrations of free BMAA was similar in all studied brain areas (i.e. cortex, hippocampus, striatum and cerebellum) whereas the hypothalamus had a higher level. The present data correspond to our previous observation of a selective uptake of radiolabelled BMAA neonatal mouse brain using autoradiographic imaging. However, the imaging study allows higher spatial resolution compared to the present UHPLC-MS/MS analysis of dissected tissues and demonstrates a heterogeneous distribution in the studied brain regions. For example, while the total concentration of BMAA in the hippocampus is not higher than the other brain regions the previous imaging study revealed a distinct uptake in the cell bodies of CA1, CA2, CA3, and dentate gyrus regions of the hippocampus^36^. The present results suggest that BMAA is taken up in the neonatal central nervous system via various amino acid transporters^30^,^36^. The developing neonatal brain has a very efficient transport of dietary amino acids^37^,^38^ that could enhance the uptake of the non-protein amino acid BMAA in the neonatal brain. Notably, the highest concentration of free BMAA occurred in the liver suggesting that there is also a selective uptake of BMAA in hepatocytes, possibly involving similar amino acid transporters. The high level of free BMAA in the rodent liver suggests that environmental sampling of animal tissues for analysis of BMAA should also include the liver.

A dose-dependent increase of protein-associated BMAA was detected in neonatal rats given BMAA. A recent time course study of radiolabelled BMAA in mouse plasma and brain demonstrated that the maximal radioactivity was measured in the acid-insoluble protein-associated fraction of brain at 4 hours and that the levels was relatively constant up to 7 days^28^. In addition, *in vitro* studies have suggested that BMAA is incorporated into the amino acid backbone of proteins^29^,^31^. It is therefore possible that the protein-associated BMAA found in the neonatal tissues may be due to a misincorporation of BMAA into proteins. It should, however, be noted that the analytical method used in the present study for the detection of protein–associated BMAA cannot exclude that BMAA is associated with tissue proteins after their synthesis via some other mechanism than through the translational machinery. TCA precipitation and thorough acetone washing of proteins may not be sufficient to remove surface associated BMAA. A recent study of BMAA protein incorporation in bacteria detected protein-association in washed and TCA precipitated proteins but not after subsequent SDS-PAGE denaturation^39^. In addition, no clear evidence for an incorporation of BMAA in newly synthesized proteins was detected by SDS denaturation in cultured human neuronal cells exposed to BMAA^40^. In contrast, Glover and coworkers report, by using a cell-free in vitro protein synthesis system, that about 50% of the protein-associated BMAA is released after SDS denaturation of the proteins but that the release of the remaining BMAA required acid hydrolysis^31^. The present study demonstrated that the protein association of BMAA in tissues with a high protein synthesis and cell turnover such as the pancreas, spleen and thymus with the exception of the liver, did not markedly exceed that of the brain tissues. However, the fraction of the tissue total BMAA that is associated to proteins was markedly higher in liver, pancreas, spleen and thymus compared to the brain and skeletal muscle^41^,^42^, with the exception of hippocampus. Since the relative rate of protein synthesis and turnover is high in the liver, pancreas, spleen and thymus^41^,^42^, this may indicate that the protein-association of BMAA in the tissues is correlated to the rate of protein synthesis.

A key issue is whether the protein-association of BMAA in the neonatal brain is a critical event for tissue-specific toxicity at this site. The level of protein-associated BMAA was significantly higher in the hippocampus compared to cortex, striatum and cerebellum. Furthermore, the percentage of the total BMAA that was associated to proteins was highest in the hippocampus. Thus tissue-specific events in the neonatal hippocampus cannot be excluded and protein-association of BMAA in the neonatal hippocampus could be a critical event resulting in the progressive neurodegeneration and intracellular fibril formation at this site in adult animals. Translational misincorporation of non-protein amino acids may generate truncated and misfolded proteins and thereby disrupt various biological functions^29^,^43^,^44^. There are, however, quality control mechanisms to degrade and eliminate misfolded proteins that may aggregate into fibrils. Misfolded proteins are normally degraded by the ubiquitin proteasomal pathways, lysosomal autophagy, or refolded by chaperons^45^. Following neonatal exposure to BMAA the expression of ubiquitin is selectively increased in the adult hippocampus indicating that ubiquitin-dependent processes are initiated in the damaged area. In addition, the negative regulator of autophagy, GLIPR-2, was exclusively detected in the damaged hippocampal region of adult rats following neonatal exposure to BMAA^21^. These observations suggest a potential aggregation of misfolded proteins at this site in adult rats.

The highest level of protein-associated BMAA was observed in the neonatal liver. De Munck and coworkers report data indicative of BMAA-induced oxidative stress in rat liver six months after BMAA exposure but conclude that the liver function should not be affected^46^. Furthermore, no acute or long-term histopathological changes have been detected in the liver after neonatal BMAA exposure^17^,^19^,^21^. This suggests that protein-association of BMAA to hepatic proteins does not induce significant cellular stress that leads to liver pathology. Interestingly, there was no increased level of protein-associated BMAA in the neonatal liver following repeated administration whereas there was a significant increase in the neonatal hippocampus. It cannot be excluded that the degradation of BMAA-associated proteins may be less efficient in neonatal hippocampus whereas the BMAA-associated proteins in the liver are more rapidly processed. Furthermore, the association of BMAA to hepatic proteins may reach a steady-state faster than the association to hippocampal proteins since the amount of free BMAA available for interaction with proteins was much higher in liver than in hippocampus. However, the animals that were given a high dose (150 mg/kg) BMAA for two days had markedly higher concentrations of protein-associated BMAA in all studied tissues compared to animals repeatedly given a low dose (5 × 40 mg/kg). This suggests that the dose protein-association relationship is not linear and that higher doses are needed for a high protein association of BMAA.

The present data confirmed our previously reported protein-association of BMAA in the liver and hypothalamus following injection of L-BMAA on PND 9 and 10 in neonatal rats^25^. The obtained data also demonstrate that the analytical method used in these two studies is highly reproducible. Similar to our earlier study, the BMAA isomer AEG was detectable at low concentrations in the protein fraction of some tissue samples. AEG was recently reported in protein-associated form in many cyanobacterial samples from fresh or marine water^47^ and in cyanobacterial crust material collected in the deserts of Qatar^48^. Although the toxicity of AEG was found to be much lower than BMAA, when comparing their median lethal concentration (LC_50_) using brine shrimp cysts as a test model, AEG was observed at a much higher concentration than BMAA in cyanobacterial samples and possible synergistic effects caused by BMAA, AEG and DAB should be considered^48^. Since cyanobacteria are primary producers in many aquatic and some terrestrial ecosystems, it would not be surprising to observe AEG in higher trophic levels in these food chains as well as in the rat chow, although the implications and mechanism behind the protein-association of AEG remain unknown.

In conclusion, the present study demonstrated that BMAA passed the neonatal blood-brain barrier and reached the rat brain. Furthermore, BMAA was associated to proteins in the neonatal brain, especially in the hippocampus. The level of protein-association in the neonatal brain was, however, significantly lower than that of the liver which is not a target organ for BMAA in neonatal rodents^17^,^19^,^21^. The protein-association of BMAA in the hippocampus and other brain areas increased following repeated administration of BMAA whereas the hepatic level of protein-associated BMAA did not increase suggesting that the rate of degradation of BMAA-associated proteins may be different in the neonatal brain and liver. However, additional evidence is needed in support of a role for protein misincorporation of BMAA in the brain for the long-term toxicity of BMAA.

## Materials and Methods

### Chemicals

Unless otherwise stated, all chemicals were obtained from Sigma-Aldrich Co. (St. Louis, MO). β-*N*-Methylamino-L-alanine (L-BMAA) hydrochloride (≥97%) was used.

### Animals and housing

Wistar rats were obtained from Taconic (Ejby, Denmark) and housed in Makrolon cages (59 × 38 × 20 cm). The animals were maintained on standard pellet food (R36 Labfor; Lantmännen, Kimstad, Sweden) with *ad libitum* access to water and housed in a temperature- and humidity-controlled environment with a 12-hour light/dark cycle (lights on at 6 a.m.). All animal experiments were approved by the Uppsala animal ethical committee and followed the guidelines of Swedish legislation on animal experimentation (Animal Welfare Act SFS1998:56) and European Union legislation (Convention ETS123 and Directive 86/609/EEC).

### Experimental design

Twelve male neonatal rats were given one daily subcutaneous injection (10 μl/g), of BMAA freshly dissolved in Hanks’ balanced salt solution at 40 mg/kg, 150 mg/kg, or vehicle for two days (postnatal days; PNDs 9 and 10, n = 4/group). The last group of rats was injected daily with 40 mg/kg BMAA for 5 days (PND 9–13, n = 4). All animals were killed by decapitation 24 hours after the last injection. Hypothalamus, cerebellum, striatum, hippocampus, cortex, liver, spleen, thymus, pancreas and skeletal muscle (*M. gracilis*) were collected and frozen immediately on dry ice (−80 °C) until sample preparation and UHPLC-MS/MS analysis.

### Quantification of free and protein-associated BMAA by UHPLC-MS/MS analysis

**Figure 3:**
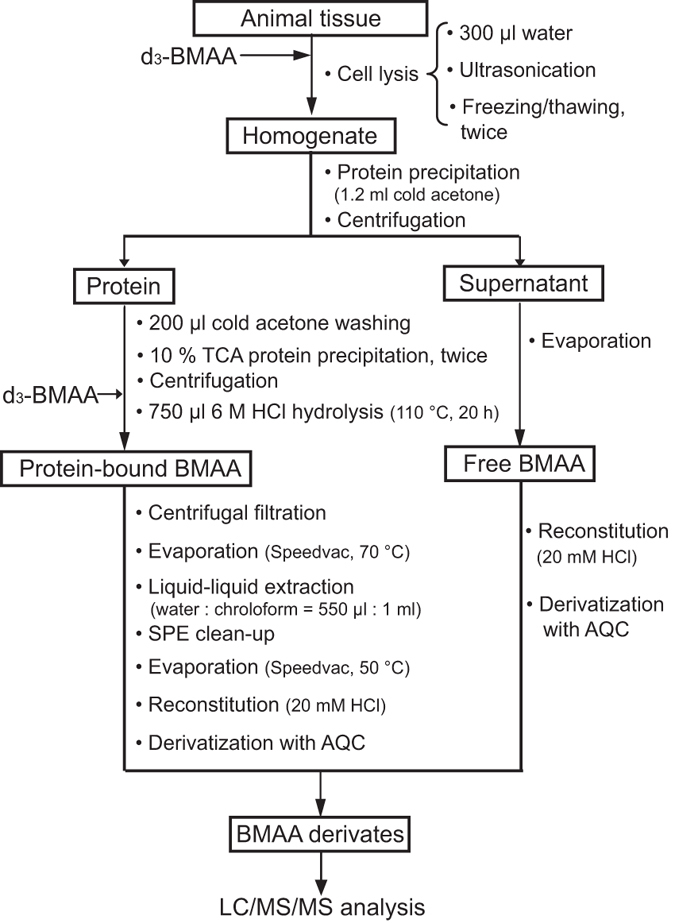
The sample preparation workflow for the detection of free and protein-associated BMAA separately using UHPLC-MS/MS analysis in tissues of neonatal rats administered BMAA.

The sample preparation was performed according to the method described in our previous study^25^. The sample preparation procedure is described in Fig. 3. Briefly, 10.0 mg (wet weight) of animal tissue was mixed with 300 μl Milli-Q water, spiked with 10 μl of the internal standard deuterium-labeled BMAA (d_3_-BMAA; 100 ng/ml) and lysed with ultra-sonication followed by freezing/thawing in liquid nitrogen. The proteins in the obtained homogenate were precipitated by adding cold acetone and keeping in the freezer at −20 °C overnight. The supernatant was separated from protein pellet after centrifugation, evaporated in a fume hood overnight, vacuum dried, reconstituted in 20 μl HCl solution (20 mM), and finally derivatized with AQC reagent (60 μl borate buffer and 20 μl AQC solution). The protein pellet was rinsed with cold acetone, reconstituted and precipitated using 10% TCA twice to remove possible free BMAA residue, and then spiked with the same amount of d_3_-BMAA as free BMAA fraction for quantification and hydrolyzed with a 6 M HCl solution (110 °C, 20 hours) to release the protein-associated BMAA. The obtained hydrolysate was filtered, cleaned up by liquid-liquid extraction and solid-phase extraction using the same protocol as described previously^49^. Finally, protein-associated BMAA was reconstituted in 30 μl HCl solution (20 mM), and derivatized with AQC reagent (60 μl borate buffer and 60 μl AQC solution). Both sample fractions (free and protein-associated BMAA) were vacuum dried and reconstituted in 30 μl 5% acetonitrile in water. An aliquot of 10 μl of the sample solution was injected into the instrument for UHPLC-MS/MS analysis.

The UHPLC-MS/MS analysis was carried out on the same LC-MS/MS instruments using the same methodology as described previously^25^. Briefly, BMAA and its structural isomers, i.e. BAMA, AEG and DAB derivates were separated on an ACCQ-TAG^TM^ ULTRA C18 column (100 × 2.1 mm, 1.7 μm particle size, Waters, Ireland) by using a UHPLC Accela-TSQ Vantage^TM^ triple quadrupole mass spectrometer (Thermo Fisher Scientific, San Jose, USA) system. A mobile phase that consists of eluent A (5% acetonitrile in water with 0.3% acetic acid) and elute B (acetonitrile with 0.3% acetic acid) was delivered at a flow rate of 200 μl/min for 10 min and then 400 μl/min for the remaining 6 min. The linear gradient elution program used was as follows: 0.0 min, 0% B; 10.0 min, 10% B; 11.0 min, 80% B; 12.0 min, 80% B; 12.1 min, 0% B; and 16.0 min, 0% B. The LC flow was directed to the waste container during the first 1 min to eliminate the borate introduced into the samples during AQC derivatization. A post-column flow of 0.3% acetic acid in acetonitrile at 600 μl/min was combined with the UHPLC flow before entering the MS ion source. The identification of BMAA was ensured by four criteria, namely 1) the chromatographic retention time; 2) precursor ion isolated in Q1 at 459.18 (*m/z*) for BMAA and its isomer AQC derivates; 3) diagnostic product ions isolated in Q3 at 258.09 (*m/z*) for BMAA and BAMA, 188.08 (*m/z*) for DAB, and 214.10 (*m/z*) for AEG; and 4) peak area ratio between general product ion at 119.08 (*m/z*) for BMAA and its three isomers, and their diagnostic product ions. The quantification of BMAA was monitored simultaneously by using the peak area ratio of the transition 459.18 > 119.08 (*m/z*) from BMAA and 462.20 > 122.10 (*m/z*) from d_3_-BMAA, and then comparing this ratio generated from a sample and from a standard solution (100 ng/ml BMAA and d_3_-BMAA) as one-point calibration. All other instrument parameters were the same as used in our previous study^49^.

## Additional Information

**How to cite this article**: Karlsson, O. *et al.* Environmental neurotoxin interaction with proteins: Dose-dependent increase of free and protein-associated BMAA (β-*N*-methylamino-L-alanine) in neonatal rat brain. *Sci. Rep.* **5**, 15570; doi: 10.1038/srep15570 (2015).

## Figures and Tables

**Table 1:** The concentration of free and protein-associated BMAA in neonatal rat tissues 24 hours after exposure to BMAA on PND 9-10.

Tissue	Dose (mg/kg)	Free BMAA (ng/mg wet tissue)	SEM	Protein-associated BMAA (ng/mg wet tissue)	SEM	Fraction protein-associated BMAA of total BMAA (%)^1^
Hippocampus	2 × 40	1.964	0.396	0.011	0.002	0.6
	2 × 150	39.809***	5.534	0.177**	0.034	0.4
	5 × 40	6.512*	1.164	0.044*	0.005	0.7
Striatum	2 × 40	4.065	0.726	ND^##^	–	ND^##^
	2 × 150	47.077***^, #^	5.239	0.048**^, #^	0.012	0.1**^, ##^
	5 × 40	9.510**	0.711	0.024**^, #^	0.002	0.2**^, #^
Cortex	2 × 40	4.551^#^	0.723	ND^##^	–	ND^##^
	2 × 150	61.839***^, #^	4.359	0.081**	0.022	0.1**^, ##^
	5 × 40	7.640	1.531	0.015**^, #^	0.006	0.2**^, #^
Hypothalamus	2 × 40	9.170	2.657	0.021^##^	0.001	0.2
	2 × 150	81.251*^, #^	20.809	0.267**	0.045	0.3
	5 × 40	14.992	5.190	0.043*	0.009	0.3
Cerebellum	2 × 40	2.692	0.336	ND^##^	–	ND^##^
	2 × 150	37.270***	3.152	0.040**^, #^	0.010	0.1**^, ##^
	5 × 40	4.347	0.753	0.010**^, ##^	0.002	0.2**^, #^
Liver	2 × 40	47.63^###^	6.66	1.23^###^	0.13	2.52^###^
	2 × 150	174.44***^, ###^	9.10	6.45***^, ###^	0.42	3.56*^, ###^
	5 × 40	66.77*^, ###^	3.77	1.03^###^	0.10	1.51*^, #^
Pancreas	2 × 40	1.143	0.182	0.011	0.001	0.94
	2 × 150	22.353***^, #^	2.796	0.137**	0.016	0.61*
	5 × 40	2.525**^, #^	0.180	0.021*^, ##^	0.003	0.81
Skeletal muscle	2 × 40	11.494^###^	0.984	0.033^###^	0.003	0.28^#^
	2 × 150	75.358***^, ##^	6.210	0.187**	0.035	0.25^#^
	5 × 40	7.867*	0.939	0.027^#^	0.002	0.34
Spleen	2 × 40	1.423	0.097	0.010	0.003	0.61
	2 × 150	11.564***^, ##^	0.121	0.075*^, #^	0.021	0.65
	5 × 40	2.060**^, ##^	0.058	0.028	0.009	1.34
Thymus	2 × 40	0.819^#^	0.125	ND^##^	–	ND^##^
	2 × 150	13.207***^, ##^	1.668	0.076**^, #^	0.006	0.6**
	5 × 40	1.435*^, ##^	0.117	0.012**^, ##^	0.001	0.8**

ND: not detectable, i.e. below 0.01 ng/mg wet tissue. Values represent mean ± SEM. *p < 0.05, **p < 0.01, ***p < 0.001 compared with the group administrated 2 × 40 mg/kg BMAA, ^#^p < 0.05, ^##^p < 0.01, ^###^p < 0.001 compared with hippocampus in respective treatment group (ANOVA followed by Student's t-test test. Chi-square test was used when groups had not detectable levels of protein bound BMAA; n = 4).

^1^Defined as concentration protein-associated BMAA/(free BMAA + protein-associated BMAA)
